# Unpeeling the layers of language: Bonobos and chimpanzees engage in cooperative turn-taking sequences

**DOI:** 10.1038/srep25887

**Published:** 2016-05-23

**Authors:** Marlen Fröhlich, Paul Kuchenbuch, Gudrun Müller, Barbara Fruth, Takeshi Furuichi, Roman M. Wittig, Simone Pika

**Affiliations:** 1 Humboldt Research Group, Max Planck Institute for Ornithology, Seewiesen, Germany; 2 Department Biology II, Ludwig-Maximilian University, Munich, Germany; 3 Centre for Research and Conservation/KMDA, Antwerp, Belgium; 4 Primate Research Institute, Kyoto University, Kyoto, Japan; 5 Department of Primatology, Max Planck Institute for Evolutionary Anthropology, Leipzig, Germany; 6 Taï Chimpanzee Project, Centre Suisse de Recherches Scientifiques, Abidjan, Côte d’Ivoire

## Abstract

Human language is a fundamentally cooperative enterprise, embodying fast-paced and extended social interactions. It has been suggested that it evolved as part of a larger adaptation of humans’ species-unique forms of cooperation. Although our closest living relatives, bonobos and chimpanzees, show general cooperative abilities, their communicative interactions seem to lack the cooperative nature of human conversation. Here, we revisited this claim by conducting the first systematic comparison of communicative interactions in mother-infant dyads living in two different communities of bonobos (*LuiKotale*, DRC; *Wamba*, DRC) and chimpanzees (*Taï South*, Côte d’Ivoire; *Kanyawara*, Uganda) in the wild. Focusing on the communicative function of joint-travel-initiation, we applied parameters of conversation analysis to gestural exchanges between mothers and infants. Results showed that communicative exchanges in both species resemble cooperative turn-taking sequences in human conversation. While bonobos consistently addressed the recipient via gaze before signal initiation and used so-called overlapping responses, chimpanzees engaged in more extended negotiations, involving frequent response waiting and gestural sequences. Our results thus strengthen the hypothesis that interactional intelligence paved the way to the cooperative endeavour of human language and suggest that social matrices highly impact upon communication styles.

“Language didn’t make interactional intelligence possible, it is interactional intelligence that made language possible as a means of communication”^1^^,p.232^

Human communication is one of the most sophisticated signalling systems in the animal kingdom and has often been used to define what it means to be ‘human^2^’. Although there is still an ongoing debate concerning which special principles form the core of this ability, most researchers would agree that it is a fundamentally cooperative enterprise^3^,^4^. The first step into this collective endeavour can already be observed in early infancy, well before the use of first words, when infants start to engage in turn-taking interactional practices embodying gestures to cooperatively share interest in an activity, event or object with other individuals^5^. One of the predominant theories of language evolution (but see for different theories^6^) thus postulates that, phylogenetically, the first fundamental steps towards human communication were not vocalisations, nor a combination of vocal and gestural signals, but were gestures alone^7^. This hypothesis stirred a considerable amount of research attention concerning general gestural abilities of our two closest living relatives, bonobos (*Pan paniscus*) and chimpanzees (*Pan troglodytes*). The resulting studies showed that both species have multifaceted gestural repertoires, which are used as flexible, intentionally produced communicative strategies in a variety of social contexts^8^,^9^,^10^. Although cooperative abilities have clearly been shown under experimental^11^,^12^ and/or natural conditions (for an overview see^13^) in both *Pan* species, some scholars have claimed that their communicative interactions lack the cooperative nature of human communication^1^,^2^. However, by combining both analytical questions and prior findings from comparative research with a conversation analysis framework (termed ‘CA-assisted comparative research’^14^), Rossano^15^ recently showed that the underlying structure of bonobo gesturing might be more similar to human conversation, and thus language, than previously thought. Conversation analysts have been intrigued with the question of how social actions can be made intelligible, since intelligibility is required to achieve mutual understanding and facilitates the successful engagement of cooperative interactions. One useful tool for addressing this question is outlining the sequential organization of social action via turns at talk^16^. The most fundamental structure in this organization is the *adjacency pair*, which can be recursively produced and extended in conversation^16^,^17^. In its minimal, unexpanded form, an adjacency pair has the following features:

- It is composed of two turns,
- by different participants,
- that are adjacently placed, and
- relatively ordered into first pair parts and second pair parts. First pair parts are actions in first position that initiate some exchange (e.g. a request: “shall we leave now?”), and second pair parts are actions in second position that are responsive to first pair parts (e.g. an answer: “yes, perfect timing”)^17^.

There are clear tendencies for a core inventory of speech acts like questions, greetings, requests, etc. (although many actions beyond the core vary), suggesting a strong universal foundation across all cultures^18^. An additional fundamental part of the infrastructure for human conversation is the temporal relationship underlying turn transitions, which is only about 200 ms on average across languages^19^. This is extraordinary if one bears in mind that latencies involved in uttering even a single word are on the order of 600 ms^20^.

In Rossano’s study, gestural sequences of two mother-infant dyads of bonobos living in captivity were investigated, with a special focus on *participation frameworks* (the signaller decides who is part of the interaction by for instance addressing her visual attention toward a recipient, expecting to turn over the communication role), cooperative *adjacency pair-like sequences* (e.g. request and answer), and temporal relationships underlying gestural performance (e.g. signaller responds immediately after a signal has been produced with a time gap of only > 0 <0.2 sec)^15^. The results showed that both dyads regularly established and engaged in *participation frameworks* and cooperative *adjacency pair-like sequences* and communicated at a speed remarkably similar to the timing of ordinary human conversation (≤0.2 sec).

Although cooperative communication between conspecifics has only been studied in bonobos thus far, other studies provide evidence for communicative differences between the two *Pan* species^10^,^21^. For instance, Pollick and de Waal^10^ found that bonobos in captivity are culturally more diverse in their gesture use and display a higher responsiveness to combinatorial signalling than chimpanzees, giving rise to the speculation that bonobos are a better model for understanding the prerequisites of human communication. Support for this bonobo-chimpanzee dichotomy also stems from other research avenues showing that bonobos are more tolerant and cooperative^11^ and outperform chimpanzees in ‘theory of mind’ tasks that require attention to social causality^22^. Recently, these differences in social cognitive abilities and social make-up have been explained not only in relation to brain regions responsible for aversive emotional stimuli eliciting fear and anxiety^23^, but also neural circuitry that may increase empathic sensitivity and prosocial behaviour^24^. The latter findings are especially important in light of shared intentionality^25^, which refers to collaborative interactions in which participants share psychological states with one another^25^,^26^. Shared intentionality has been suggested as the driving force and “the small psychological difference” in human cognitive evolution that paved the way for the cooperative endeavor of language^26^,^27^.

The aim of the present study was twofold: First, we revisited the claim that communicative interactions of our closest living relatives, bonobos and chimpanzees, lack the cooperative nature of human communication^1^,^2^. Second, we investigated whether bonobos are the better model species for understanding the precursors of human communication^10^.

To target these aims, we tested and expanded some of the parameters used by Rossano^15^ *in situ* (i.e. in bonobos and chimpanzees living in their natural environments) by focusing on the core niche in which communication is learned—face-to-face interactions of mother-infant dyads. Since it recently had been emphasized that due to relatively high degrees of behavioural plasticity both *Pan* species show considerable inter-site variation (for an overview see^28^), we investigated communicative interactions of two bonobo and two chimpanzee communities at four different sites and locations. In the case of behavioural differences between communities, this study design then offered the possibility to distinguish between within- versus between-species differences^29^. Data were collected at *LuiKotale,* Salonga National Park, Democratic Republic of the Congo (DRC), *Wamba*, Luo Scientific Reserve, DRC, *Kanyawara*, Kibale National Park, Uganda (*Pan troglodytes schweinfurthii*) and *Taï South*, Taï National Park, Côte d’Ivoire (*Pan troglodytes verus*). We focused on the single communicative function of mother-infant joint travel, since previous studies suggested that this context is a promising candidate for the occurrence of frequent turn-taking sequences to achieve a joint goal (leaving a location)^15^,^30^. The following criteria were analysed: (i) *establishment of participation frameworks* (i.e. the initiator establishes for instance via gaze, body direction who is addressed and will be part of the communicative interaction before performing a gestural signal) by focusing on the parameters of gaze, body orientation and initiation distance; (ii) *adjacency pair-like sequences* (i.e. analysing gestures and their respective responses) by examining the parameters number of gestural requests and their respective responses (so called gesture-response pairs) leading to joint travel and response waiting (i.e. signaller pauses after the signal has been produced for at least two seconds waiting for a response) after each solicitation; and (iii) the *temporal relationships* between joint-travel-initiating behaviour and response, by focusing on the parameters of delayed (> 2 sec), immediate (≥ 0 < 2 sec) and overlapped responses (< 0 sec, for further details see also methods).

Overall, the results of the present study provide evidence that bonobo and chimpanzee mother-infant dyads frequently engage in cooperative turn-taking sequences, and thereby exhibit many similarities to human social action during conversation. The two species differed significantly with regard to some of the examined parameters: While gaze, close initiation distance and fast-paced responses characterised mother-infant joint travel interactions in bonobos, chimpanzees exhibited a higher number of gesture-response pairs, a higher frequency of response waiting and more delayed responses. By using a combination of methods thus far predominantly applied to human social interactions^16^,^17^, with an unprecedented within-group, between-group and between species comparison of apes living in natural environments, we show that (a) cooperative communication has a more ancient evolutionary origin than previously thought, and (b) social matrices strongly influence communicative preferences and styles. Our study strengthens the view that human communication represents an ensemble of layers of abilities of different types and different antiquity, with precursor adaptations in turn-taking behaviours in which gestures (and possibly other signals) are embedded^31^.

## Results

For our analyses, we distinguished gestures fulfilling only one key characteristic of intentional communication^5^,^30^ (single-criteria or SC-gestures) from those that conformed to several key characteristics of intentional communication (multiple-criteria or MC-gestures). These criteria included parameters such as sensitivity to recipients’ attentional states, response waiting and goal persistence^5^,^30^ (for further details see methods and video clips 1–4 in the SM).

The coding of the data set resulted in a total of 400 SC-gestures in bonobos (*LuiKotale*: N = 166; *Wamba*: N = 234) and 637 in chimpanzees (*Kanyawara*: N = 274; *Taï South*: N = 363). Concerning MC-gestures, we found a total of 313 gestures in bonobos (*LuiKotale*: N = 152; *Wamba*: N = 161) and 612 in chimpanzees (*Kanyawara*: N = 361; *Taï South*: N = 251). For detailed descriptions of the gestures used in joint travel interactions, see Supplementary Material, Table S1.

### Establishment of participation frameworks

We investigated whether mother-infant dyads established *participation frameworks* before the start of joint travel by analysing the parameters of gaze, body orientation and initiation distance both within and between species. To test the extent to which species and site but also the variables of dyadic role and infant age influenced these three parameters, we used Generalized Linear Mixed Models (GLMM^32^) for each parameter. Overall, the test predictors had a clear impact in all three models (likelihood ratio tests comparing null and the full model for gaze: *χ*^2^ = 16.8, *df* = 8, *p* = 0.03; body orientation: *χ*^2^ = 178.9, *df* = 8, *p *< 0.001; distance: *χ*^2^ = 26.1, *df* = 8, *p* = 0.001). The behavioural differences that we found provided evidence for species differences, but not for within-species variability (likelihood ratio tests comparing null and the reduced model lacking site effects; gaze: *χ*^2^ = 0.2, *df* = 4, *p* = 0.99; body orientation: *χ*^2^* *< 0.001, *df* = 4, *p* = 1; distance: *χ*^2^ = 0.009, *df* = 4, *p* = 0.99).

**Figure 1:**
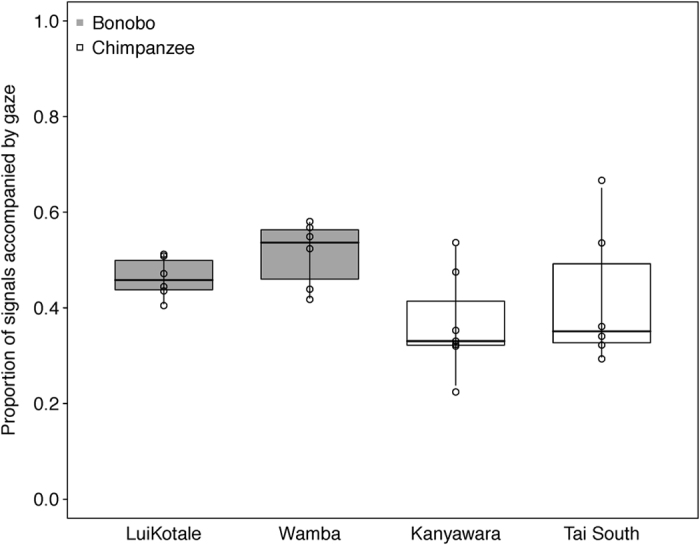
Proportion of gaze before signal initiation in bonobo (grey) and chimpanzee (white) mother-infant dyads as a function of study site. Dots represent mean proportions per dyad. Indicated are median (horizontal lines), quartiles (boxes) and percentiles (2.5 and 97.5%, vertical lines).

**Figure 2:**
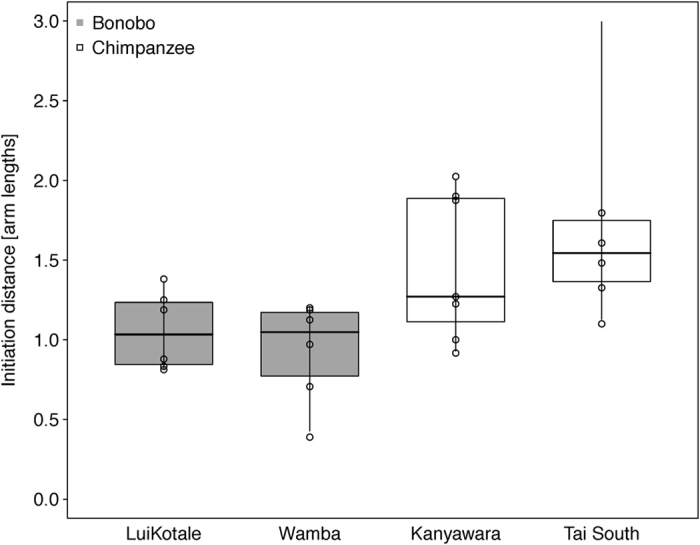
Distance between bonobo (grey boxes) and chimpanzee (white boxes) mother-infant dyads at joint-travel-initiation as a function of study site. Dots represent average initiation distances per dyad. Indicated are median (horizontal lines), quartiles (boxes) and percentiles (2.5 and 97.5%, vertical lines).

Concerning gaze, species was the only significant predictor, with bonobos accompanying more initiatory behaviours with gaze than did chimpanzees (estimate ± SE = −0.485 ± 0.155, *χ*^2^ = 8.288, *df* = 1, *p* = 0.004; see Fig. 1). With respect to body orientation, we found a significant interaction between dyadic role and infant age (role*between-infants age: −0.483 ± 0.128 *χ*^2^ = 16.307, *df* = 1, *p* <0.001). While infants oriented more towards the recipient the older they were, there was no effect for mothers with increasing age of their infants. Additionally, chimpanzees more frequently oriented their bodies toward the recipient before signalling than did bonobos (0.543 ± 0.179, *χ*^2^ = 4.872, *df* = 1, *p* = 0.027). Chimpanzee dyads initiated joint travel from a significantly larger distance than did bonobo dyads (0.426 ± 0.103, *χ*^2^ = 7.382, *df* = 1, *p* = 0.007; see Fig. 2). Furthermore, initiation distance significantly increased with infant age in both species (between-infants age: 0.145 ± 0.041, *χ*^2^ = 9.919, *df* = 1, *p* = 0.002). No other effects in the three models reached significance (see Table 1; sections 1.a-1.c).

### Adjacency pair-like sequences

To examine whether mother-infant dyads form successful *adjacency pair-like sequences* to initiate joint travel, we investigated two parameters: number of gesture-response pairs and response waiting (i.e. signaller pauses at the end of a given signal for at least two seconds waiting for a response; see also methods). To test the influence of species, site, dyadic role and infant age on these three parameters, we used Generalized Linear Mixed Models (GLMM^32^) for each of them. The test predictors had a clear impact on both models (likelihood ratio tests comparing the null and the full model for gesture-response pairs: *χ*^2^ = 20.9, *df* = 8, *p* = 0.007; response waiting: *χ*^2^ = 42.2, *df* = 8, *p *< 0.001). The differences we found could only be ascribed to inter-species, rather than intra-species, variability (gesture-response pairs: *χ*^2^* *< 0.001, *df* = 4, *p* = 1; response waiting: *χ*^2^* *< 0.001, *df* = 4, *p* = 1).

**Figure 3:**
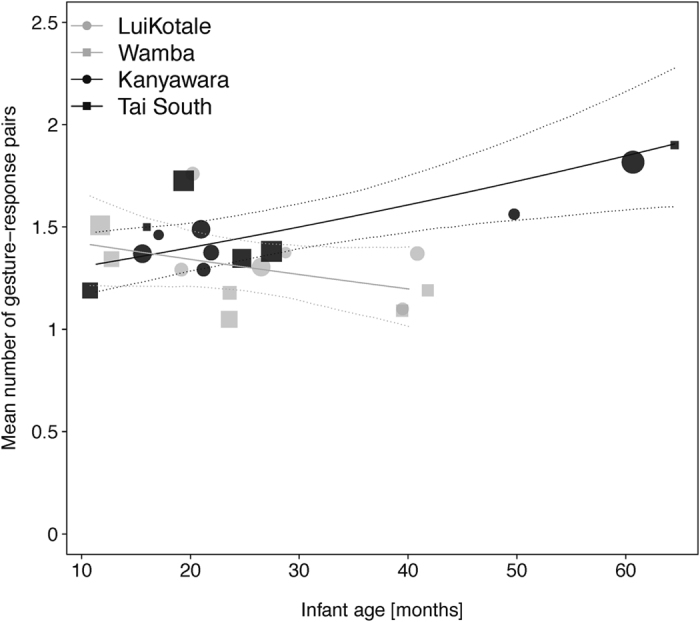
Count of gesture-response pairs to achieve joint travel in bonobo (grey symbols) and chimpanzee (black symbols) mother-infant dyads of four different communities as a function of infant age. Depicted are average numbers, separately for each dyad against its mean infant age. The area of the symbols corresponds to the sample size per dyad; the solid and dashed lines represent the fitted model and confidence intervals are based on all other covariates and factors centred to a mean of zero.

**Figure 4:**
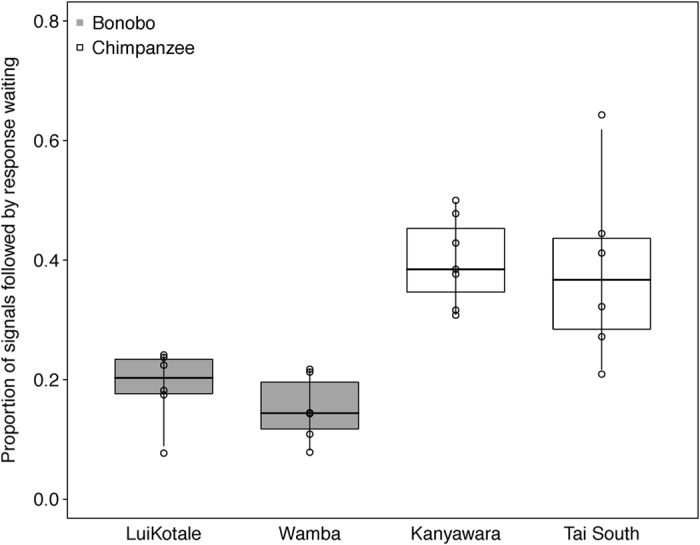
Proportion of signals followed by response waiting in bonobo (grey boxes) and chimpanzee (white boxes) mother-infant dyads as a function of study site. Dots represent mean proportions per dyad. Indicated are median (horizontal lines), quartiles (boxes) and percentiles (2.5 and 97.5%, vertical lines).

Regarding the number of gesture-response pairs, a significant interaction between species and between-infants age was found (0.179 ± 0.071, *χ*^2^ = 6.381, *df* = 1, *p* = 0.012; see Fig. 3). While in bonobos the number of gesture-response pairs decreased, it increased with age in chimpanzees. In addition, mothers produced significantly more gesture-response pairs than infants (0.18 ± 0.078, *χ*^2^ = 5.447, *df* = 1, *p* = 0.02). For the parameter of response waiting, we found a significant interaction between dyadic role and between-infants age (−0.433 ± 0.114, *χ*^2^ = 14.856, *df* = 1, *p *< 0.001). While infants were more likely to wait for a response with increasing age, the age of infants did not influence the occurrence of response waiting in mothers. In addition, chimpanzees were more likely to wait for a response by the recipient than bonobos (0.893 ± 0.173, *χ*^2^ = 19.845, *df* = 1, *p *< 0.001; see Fig. 4). No other effects in the two models reached significance (Tables 1, sections 2.a–2.b).

### Temporal relationships between signal and response

We examined whether there were inter- or intra-species differences concerning the timing of a given response after a travel-initiating gesture (i.e. SC-gestures and MC-gestures) had been produced. We differentiated between immediate (Δt [start response – end initiatory gesture] > 0 < 2 s), delayed (start response ≥ 2 s after end initiatory gesture) and overlapping responses (Δt [start response – end initiatory gesture] < 0; for detailed definitions see methods). Bonobos predominantly produced overlapping (N = 200, 47.1%; *LuiKotale*: N = 89, 46.8%; *Wamba*: N = 111, 47.4%) and immediate responses (N = 143, 33.5%; *LuiKotale*: N = 59, 31.1%; *Wamba*: N = 84, 35.9%), but relatively low frequencies of delayed responses (N = 81, 19.4%; *LuiKotale*: N = 42, 22.1%; *Wamba*: N = 39, 16.7%). In contrast, chimpanzees produced approximately equal proportions of overlapping (N = 291, 32.3%; *Kanyawara*: N = 138, 30.5%; *Taï South*: N = 153, 34.1%), immediate (N = 289, 32.1%; *Kanyawara*: N = 157, 34.7%; *Taï South*: N = 132, 29.4%), and delayed responses (N = 321, 35.6%; *Kanyawara*: N = 157, 34.7%; *Taï South*: N = 164, 36.5%). For example see video clips 1–4 in the Supplementary Material. To test the extent to which species, site, dyadic role and infant age influenced the three response types, we used Generalized Linear Mixed Models (GLMM^32^) for each parameter. The test predictors had a clear impact on the occurrence of overlapping and delayed responses, but not on the occurrence of immediate responses (overlapping response: *χ*^2^ = 29.6, *df* = 8, *p* = 0.005; immediate response: *χ*^2^ = 5.6, *df* = 8, *p* = 0.694; delayed response: *χ*^2^ = 35.9, *df* = 8, *p *< 0.001). The likelihood ratio test comparing the full models with the reduced models revealed that behavioural differences mirrored species differences but not within-species variability (overlapping: *χ*^2^ = 0, *df* = 4, *p* = 1; delayed: *χ*^2^* *< 0.001, *df* = 4, *p* = 1; see also Supplementary Material, Figure S1).

**Figure 5:**
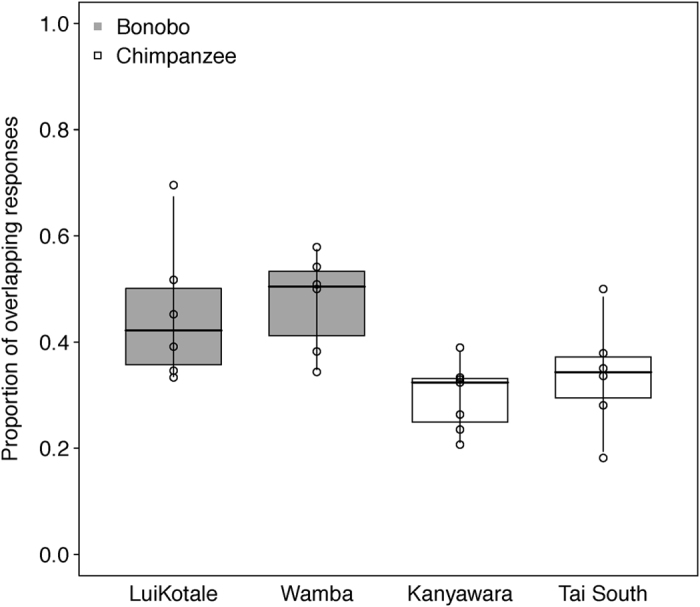
Proportion of overlapping responses in bonobo (grey boxes) and chimpanzee (white boxes) mother-infant dyads as a function of study site. Dots represent means per dyad. Indicated are median (horizontal lines), quartiles (boxes) and percentiles (2.5 and 97.5%, vertical lines).

**Figure 6:**
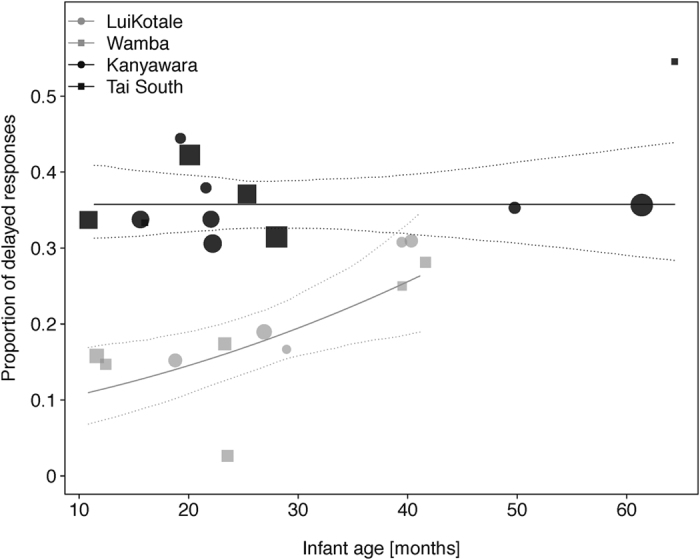
Proportion of delayed responses in bonobo (grey symbols) and chimpanzee (black symbols) mother-infant dyads of four different study sites as a function of infant age. Depicted are proportions, separately for each dyad against its mean infant age. The area of the symbols corresponds to the sample size per dyad; the solid and dashed lines represent the fitted model and confidence intervals are based on all other covariates and factors centred to a mean of zero.

Overlapping responses were significantly more frequent in bonobos (−0.65 ± 0.127, *χ*^2^ = 11.656, *df* = 1, *p *< 0.001; see Fig. 5) than in chimpanzees. Furthermore, infants of both species were more likely to use overlapping responses than were mothers (0.382 ± 0.135, *χ*^2^ = 8.256, *df* = 1, *p* = 0.004). Overlapping responses were significantly more frequent in dyads with younger infants than in dyads with older infants (−0.124 ± 0.063, *χ*^2^ = 3.953, *df* = 1, *p* = 0.047), irrespective of species. Concerning delayed responses, we found a significant interaction between species and between-infants age, with chimpanzees producing more delayed responses across infant ages than did bonobos (−0.51 ± 0.187, *χ*^2^ = 7.564, *df* = 1, *p* = 0.006; see Fig. 6). Mothers of both species were also more likely to produce delayed responses than their infants (−0.484 ± 0.137, *χ*^2^ = 12.416, *df* = 1, *p *< 0.001). No other effect in the models reached significance (see Table 1, sections 3.a–3.b).

## Discussion

The aim of the present study was twofold: First, we wanted to revisit the claim that human cooperative communication evolved as part of a larger, uniquely human, adaptation for cooperation and cultural life in general^1^,^2^. Second, we examined whether bonobos are the better model species for understanding the prerequisites of human communication^10^. To do so, we investigated whether bonobos and chimpanzees, both of which engage in general cooperative activities^11^,^12^,^13^, use distinct features characteristic of human social action in conversation^16^,^17^. By taking into consideration intra- and inter-species variability and by focusing on the mother-infant dyad, our results showed that all observed dyads across groups frequently engaged in turn-taking sequences to negotiate joint travel. They established *participation frameworks* via gaze, body orientation and the adjustment of initiation distance, and they used *adjacency pair-like sequences* characterized by gesture-response pairs and response waiting. Regarding *temporal relationships* between signals and responses, we found that mother-infant dyads of both species used the whole spectrum of responses, including immediate, overlapping and even delayed responses. Immediate responses match the temporal relations between turns in human speech consisting of relatively little cultural variation (e.g. overall cross-linguistic median of 100 ms, ranging from 0 ms in the English and Japanese culture, for instance, to 300 ms in the Danish and Lao culture)^19^. Our findings therefore support and expand the results of Rossano^15^, by demonstrating that gestural exchanges observed in mother-infant dyads of bonobos and chimpanzees are often very similar in timing to human action in conversation and embody the most crucial features of human cooperative conversation^4^,^27^: These gestural exchanges are bidirectional coordination devices, comprising the two implicit roles of signaller and recipient. In learning to use these gestures, individuals learn to play and to comprehend both roles no matter which role they are performing (soliciting to leave a location or being solicited to leave a location). Although we recently showed that chimpanzee mothers and their infants differ in many of the gesture types employed to initiate joint travel^30^, gestures shared by mothers and infants might involve role-reversal imitation (i.e. when one uses a gesture toward others the way others have used this gesture toward oneself), but also take the other’s perspectives on the event of joint travel. Furthermore, to reach the joint goal (of leaving a location), signallers made efforts to communicate in ways that were comprehensible to the recipient, for instance by combining initiatory behaviours with gaze and orienting the body to recipients. In addition, they seemed to ‘clarify’ the intended goal by using several *adjacency pair-like sequences* composed of the same or different gestures, when the first communicative attempt had not been successful. Turn-taking sequences of pre-linguistic human children go a step further in that recipients ask for clarification when needed and employ ‘negotiation of meaning’^33^. Furthermore, the rapid turn-taking in human conversation involves indefinite varying contents of turns, multi-modal deployment of vocal and gestural signals, and also seems without parallel, given the sheer amount of time and effort invested in communication^31^ (but see for nonhuman primates and birds^34^,^35^).

Overall, our findings strengthen a recent proposal by Levinson and Holler^31^ emphasizing the role of turn-taking behaviour for evolutionary scenarios of human language. They suggest that human language, despite its tight integration of speech and gesture, is a system composed of layers of abilities of different types and different antiquity. Thus, unpeeling the layers should enable us to understand the evolution of human language from an original rapid exchange of gestural or vocal material, into a system where the complexity of the linguistic and gestural material that is expressed in relatively short bursts has grown to the very limits that human cognition can process^31^.

In sum, sequentially organized, cooperative social interactions are not simply by-products of individuals living in human enculturated environments^15^, but play a crucial role in communicative exchanges of mother-infant dyads of bonobos and chimpanzees living under active selection pressures. These results challenge the human-ape divide, which suggests that human cooperative communication evolved as part of a larger adaptation of humans’ species-unique forms of cooperation^2^ ratcheted via existing and simpler components of primate cognition, such as group action and manipulative communication^26^. Our findings indicate that cooperative communicative interactions seem to play a crucial role in mother-infant dyads of bonobos and chimpanzees and, more generally, in nonhuman animals, for which shared goals and relatively low levels of competition prevail. Similarly to the universally organized social-interaction matrix of human conversation^19^, the results suggest that our closest living relatives have a strong universal infrastructure underlying their gestural interactions, which serves to minimize gaps and overlaps and allows for efficient information exchange. Further research on the methods and model species commonly used to draw inferences about evolutionary precursors to human communication is warranted (see for recent developments in other areas of cognitive ethology^36^), to enable (i) higher sensitivity to the social characteristics and/or ecology of a given species and (ii) a vital understanding of the structure and cognitive complexity underlying turn-taking sequences^14^ and communicative exchanges such as vocal alternations^34^ and duetting^37^.

Concerning our second aim of examining whether bonobos are the better models for precursors to human communication, a comparison of the investigated parameters showed behavioural differences between species, but not within species. Specifically, bonobo dyads (i) accompanied their signals more frequently with gaze, (ii) stayed in closer spatial proximity to each other for mother-infant coordination, and (iii) preferred to use overlapping and immediate responses. In contrast, chimpanzee dyads (i) were more likely to orient their bodies toward a recipient before signalling, (ii) showed a higher number of gesture-response pairs and response waiting, (iii) displayed overall more ‘communicative persistence’ to obtain the desired goal of joint travel, and (iv) used all three response tempi with relatively similar frequencies.

Three hypotheses may account for these observations. First, differences in communicative patterns may be explained by differences in ecological environments. For example, habitat characteristics such as thickness and growth of terrestrial herbaceous vegetation (THV) may differ considerately between the sites, resulting in different degrees of visibility and thus communication space and eye contact. Although THV is clearly more prevalent in bonobo habitats than in chimpanzee habitats^38^, differences in THV might also exist between the two chimpanzee habitats, resulting in relatively higher levels of visibility at *Kanyawara*^38^ compared to *Taï South*^39^. If this hypothesis were true, we would have expected to find differences in communicative behaviours between the *Kanyawara* and the *Taï South* community *and* between bonobos and chimpanzees in general. This does not accord with our observations.

Second, differences in communication styles between bonobos and chimpanzees are a by-product of the studied age range, particularly because chimpanzee infants may generally develop more quickly than do bonobo infants. For instance, Kuroda^40^ suggested that growth rates of bonobos and chimpanzees differ considerably, such that bonobos undergo a slower development of (i) spatial independence, (ii) locomotor skills (e.g. climbing, walking quadrupedally, riding on mother’s back), and (iii) social interactions with conspecifics (e.g. approaching, playing). This proposed delay in general development in bonobos may also have a crucial impact on the speed of communicatory skill development. If this hypothesis were true, we would have expected to find that age had a significant impact on the investigated parameters, with bonobo infants showing certain parameters such as use of gaze, adjustment of body orientation and response waiting, as well as overlapping responses, significantly later than chimpanzee infants. However, this was not the case. Body orientation and initiation distance were the only parameters for which a developmental effect was found, with increases of adjustment of body orientation towards mothers and initiation distance with age in both bonobo and chimpanzee infants. Overall, chimpanzee dyads initiated joint travel from larger distances than did bonobo dyads. These results are in line with findings of de Lathouwers and colleagues^41^, who showed that immature chimpanzees spend more time at larger distances from their mothers than do immature bonobos. Our study confirms that chimpanzees indeed develop spatial independence more quickly than do bonobos.

Third, bonobos and chimpanzees might employ different communication styles. Consistent with this hypothesis and based on our investigated parameters, bonobos and chimpanzees could be characterized by two clearly distinguishable communication styles accompanied by different temporal relationships: Bonobos frequently combined their communicative signals with gaze while in close proximity to the addressee, and they often used speedy responses. The underlying temporal relationships often matched those underlying human turn transition during speech^19^, with predominantly single *adjacency pairs* but also recipients responding before signals had been fully articulated. Chimpanzees, on the other hand, adjusted their body orientation toward recipients and used a generally slower mode of communication that involved more gesture-response pairs, higher frequencies of response waiting and delayed responses. Bonobo communication thus seems to resemble a subtle dance coined of flowing movements by signallers and recipients, while chimpanzee communication is structured with temporally separated and clearly recognizable units such as signal, pause and response. Chimpanzee signalling mirrors the structure of other social interactions, such as aggressive and grooming interactions, which are also characterized by typical negotiation sequences^42^,^43^, thereby demonstrating the significance of clearly structured interactions in chimpanzee society.

While future studies with additional age classes, communicative functions and dyads are of course mandatory, our study provides the first evidence that mother-infant dyads of bonobos and chimpanzees living in their natural environments employ different communication styles to convey the same message. Moreover, if certain communicative patterns are already observable in mother-infant coordination, the first step of co-regulated social interaction^44^, it is likely that these patterns are also crucial for general communication abilities of the species. Thus, generalization to behaviours of other dyads of a given community may be possible to some extent.

Although the long-standing bonobo-chimpanzee dichotomy has been challenged by new data emphasizing intra-species over inter-species variability^28^, bonobos and chimpanzees still seem to differ considerably concerning distinct characteristics of their social matrices. Males are more influential in chimpanzee society than in bonobo society^45^,^46^, with male chimpanzees heavily competing within their communities to gain indirect and direct fitness benefits^13^,^46^. This competition results in linear dominance hierarchies, male harassment and male-female dominance, but also strong social bonds and cooperative behaviour between males in the form of short- and long-term alliances (e.g. in the form of coalitionary behaviour, grooming, meat sharing and border patrols^13^,^46^). High levels of aggression, including lethal attacks, characterize intergroup encounters in chimpanzees, and infanticide has been observed within and between communities^47^. In contrast, bonobo society is characterized by co-dominance between the sexes, prolonged mother-son relationships^45^, and strong bonds between unrelated females^45^,^48^, resulting in a more flexible choice of coalition partners. Although between-group encounters in bonobos are usually friendly and peaceful^49^, there is anecdotal evidence of attempts of infanticide by males^50^ but also females^51^. Given the gregariousness of bonobo females, the threat of female infanticide could explain the need for close range communication between mothers and their dependant offspring.

It has been argued that species-specific social matrices and behaviours have been evolutionarily shaped by the distinctive morphology, connectivity and molecular biology of brain regions and pathways involved in social and environmental appraisal of threats and vigilance and control of emotional responses^23^,^24^. For instance, bonobos have more gray matter in the dorsal amygdala and a larger pathway linking the amygdala with the ventromedial prefrontal cortex (VMPFC)^24^. This neural circuitry has been implicated in both top-down control of aggressive impulses and bottom-up biases against harming others, as well as increased empathic sensitivity and prosocial behaviour^24^. In addition, bonobos have approximately twice the density of serotonergic axons in the amygdala compared to chimpanzees^51^, contributing to appraisal of the emotional context and significance of the environment^52^. These differences in neural circuitry are in line with recent experimental findings showing that bonobos tend to exhibit more cautious temperaments^53^, reduced ‘emotional reactivity’^11^, and greater tolerance when competing over food-resources^36^. The results of our study suggest that crucial features characterizing human communication, such as gaze and anticipation of recipients’ behaviour^2^,^5^, may be more significant in bonobo than in chimpanzee communication. Bonobos appear to exhibit a higher social awareness of the communicative situation and the anticipated meaning of a given signal, strengthening recent results demonstrating a bonobo-chimpanzee divergence in tasks requiring attention to social causality^22^.

Bonobos may therefore represent the most representative model for understanding the prerequisites of human communication^10^. However, additional analyses of the communicative and cognitive abilities of our closest living relatives are compulsory for a complete understanding of the impact of social and possibly cultural matrices on communication styles and tendencies. In addition, examples of convergent evolution in distantly related species can provide clues to the types of problems that particular communicative mechanisms are ‘designed’ to solve^54^,^55^. We thus hope to inspire future research that not only incorporates additional dyads and contexts, but also conducts taxonomically informed comparisons of species engaging in turn-taking behaviour during general interactions and communicative exchanges.

In sum, our results provide substantial evidence that the two primary model species for the origins of human behaviour, bonobos and chimpanzees, differ in their communication styles. While bonobos seem to anticipate and respond to signals before they have been fully articulated, chimpanzees engage in more time-consuming communicative negotiations. Both species use sequentially organized, cooperative social interactions to engage in a joint enterprise: Leaving together to another location. Their communicative interactions thus show the hallmarks of human social action during conversation and suggest that cooperative communication arose as a way of coordinating collaborative activities more efficiently. Our results strengthen a recent proposal by Levinson and Holler^31^ suggesting that the apparent gulf between animal and human communication may be bridged by looking for precursors adaptations to human language in turn-taking interactions.

## Methods

### Study sites and subjects

The study was conducted at two communities of bonobos (*LuiKotale* at the fringe of Salonga National Park, DRC; *Wamba* in the Luo Scientific Reserve, DRC) and two communities of chimpanzees (*Kanyawara* in Kibale National Park, Uganda; *Taï South* in Taï National Park, Côte d’Ivoire). Detailed descriptions of the study areas can be found in Hohmann and Fruth^56^ for *LuiKotale,* Kano^57^ for *Wamba,* Wrangham and colleagues^58^ for *Kanyawara* and Boesch and Boesch-Achermann^39^ for *Taï South*. The communicative behaviour of bonobos was observed at *LuiKotale* by P.K. and a trained field assistant from April to November 2012 and from April to July 2013. A second trained field assistant collected data at *Wamba* from September 2012 to February 2013. The behaviour of chimpanzees was observed by M.F. during four study periods between October 2012 and June 2014 (*Kanyawara*: Mar–May 2013, Mar–Jun 2014; *Taï South*: Oct–Dec 2012, Oct–Dec 2013). During the study periods, the number of community members varied between 35 and 40 at *LuiKotale*, around 31 at *Wamba,* between 53 and 56 at *Kanyawara* and between 26 and 33 individuals at the *Taï South* community. Bonobos and chimpanzees at all four sites were well habituated to human observers and have been studied on a longitudinal basis since 2003 in *LuiKotale*^56^, 1973 in *Wamba*^57^, 1987 in *Kanyawara*^58^ and 1979 in *Taï South*^39^. It was therefore possible to observe the community members during dawn-till-dusk follows and to collect high-quality video and audio footage. In addition, we had access to long-term data on demography and relatedness for all four field sites. We observed communicative interactions between mothers and their youngest dependent offspring in a total of 12 bonobo dyads and 13 chimpanzee dyads: Six dyads were observed at *LuiKotale*, six at *Wamba,* seven at *Kanyawara* and six at *Taï South*. The age of the offspring ranged from ten to 56 months in bonobos and nine to 69 months in chimpanzees (see Table 2 for detailed information on subjects and data sets).

### Data collection

We used a focal behaviour sampling approach^59^, while maintaining a record of the frequency with which a particular dyad had been observed. In situations where we could choose which of several dyads to film, we targeted those individuals previously sampled least often. All social interactions involving mothers and infants (i.e. mother-infant as well as mother-conspecific and infant-conspecific interactions) that were judged to have any potential for communicative interactions were recorded using a digital High-Definition camera (Canon Legria HF M41) with an externally attached unidirectional microphone (Sennheiser K6). During approximately a total of 2200 hours of observation (1033 hours for bonobos; 1189 hours for chimpanzees), we collected a total of 238.5 hours of video footage on the communicative behaviour of 12 bonobo (*Wamba*: 41.5 h; *LuiKotale*: 51.9 h) and 13 chimpanzee (*Taï South*: 73.4 h; *Kanyawara*: 95.5 h) mother-infant dyads (mean ± SD per dyad = 9.5 ± 3.5 h; see Table 2 for further details on data collection). However, since this paper focuses only on the communicative context of joint travel, our analysis is based on a total of 319 bonobo and 410 chimpanzee video recordings of mother-infant joint travel interactions (mean recordings per bonobo/chimpanzee dyad: 26.6/31.9; see Supplementary Material, Table S1). In addition, we included five joint-travel interactions that were recorded with a Pocket PC (HP iPAQ rx1959), resulting in a total of 415 interactions for chimpanzees.

### Coding of behaviours

To establish the repertoires employed to initiate joint travel and to enable subsequent analyses, a total of 729 high-quality video files of mother-offspring joint-travel-initiations (i.e. carries with clear visibility of joint-travel-initiating behaviours) were coded using the program Adobe Premiere Pro CS4 (version 4.2.1). Behavioural definitions were based on established ethograms of bonobo^57^ and chimpanzee behaviour^60^. The coding scheme was designed by using parameters developed in previous work on great ape gesturing^30^,^61^. We only coded successful, agent-initiated joint travel interactions, meaning those interactions leading to infants leaving a location attached to their mothers in a dorsal or ventral carry position. We differentiated between joint-travel-initiations via intentionally produced gestures showing more than one key characteristic of intentional communication and gestures showing only one key characteristic of intentionality, which are respectively called multiple-criteria gestures (MC-gestures) and single-criteria gestures (SC-gestures). Gestures were defined as directed, mechanically ineffective movements of the body or body postures that elicited (“requested”) a voluntary response by the recipient^62^. The following key characteristics of intentional communication were measured^5^,^30^:

#### Sensitivity to the attentional state of the recipient

The signaller shows signs of being aware of the recipient’s state of attention, e.g. by using visual gestures only when the recipient is looking.

#### Response waiting

The signaller pauses at the end of the production of a signal and waits for at least two seconds expecting a response, while maintaining visual contact with the recipient.

#### Apparent satisfaction of signaller

The signaller’s communication ceases when the apparent goal has been met by the recipient (leaving the location together).

#### Goal persistence

The signaller elaborates her signalling when thwarted, e.g. by repeating and exaggerating the signal or by using a different means of communication.

##### Establishment of participation frameworks

We examined three different parameters:

#### Gaze

Signaller looks at the recipient while executing the gesture.

#### Body orientation

Recipient is positioned directly in front of and in the visual field of the signaller.

#### Initiation distance

Physical distance (in arm lengths) between signaller and recipient at first joint-travel-initiating gesture.

##### Adjacency pair-like sequences

To investigate whether bonobos and chimpanzees use adjacency pair-like sequences, we focused on gesture-response pairs and response waiting (see paragraph above for definition) after each solicitation gesture. Regarding gesture-response pairs, we analysed the gestures and gestural bouts that included single gestures or sequences, separated by periods of response waiting lasting more than a second and followed by a recipient’s response.

##### Temporal relationships between turns

We assessed the temporal relationships between signals and their respective responses by differentiating between three types of responses (although see for exact times Supplementary Material, Figure S1):

#### Immediate response

The joint-travel-initiating behaviour is followed by a response of the recipient (Δt [start response – end of initiatory gesture] >0) less than two seconds after the behaviour has been articulated.

#### Delayed response

The joint-travel-initiating behaviour is followed by a response of the recipient more than two seconds after the behaviour has been articulated (end of initiatory gesture).

#### Overlapping response

The joint-travel-initiating behaviour (SC- or MC- gesture) is followed by a response of the recipient either before the behaviour has been fully executed (Δt [start response – end initiatory gesture] <0), or less than one second after it has been fully articulated. The full articulation was deciphered by the observer when a given gesture was followed by the immediate reaction (on behalf of the recipient) or response waiting (on behalf of the signaller).

For each gesture used to solicit joint travel, we coded the interaction role of the actor (2 levels: mother, infant), infant age (range = 9–69 months), infant sex (2 levels: female, male), and mother’s parity (range = 1–5 offspring). Fifteen per cent of all mother-infant interactions were coded for accuracy by a second observer and tested using the Cohen’s Kappa coefficient to ensure inter-observer reliability^59^ with the following results: A ‘very good’ level of agreement for initiatory gesture type/gaze (κ = 0.80), joint-travel-initiator (κ = 0.84), orientation and distance to recipient (κ = 0.89), gesture-response pairs (κ = 0.89), and temporal relationships including response waiting (κ = 0.85).

### Analyses

To test the extent to which species and/or site, but also other parameters such as dyadic role and infant age, influenced i) the *establishment of participation frameworks* before signalling (response variables: gaze, body orientation, initiation distance), ii) successful *adjacency pair-like sequences* (response variables: number of gesture-response pairs, response waiting), and iii) the timing of signal and response (response variables: immediate response, delayed response and overlapping response), we used Generalized Linear Mixed Models (GLMM^32^) with a binomial error structure and logit link function. For the number of gesture-response pairs and distance, we used a Poisson error structure and log link function. We included species, dyadic role and infant age as our key test predictors. Since age varied considerably between infants, we used the method of within-subject centering^63^ to determine whether the effect of infant age was particularly relevant within and/or between infants. Hence, we included in the model the average age of each infant (constant across all data points of the respective mother-infant pair; ‘between-infants age’) and the difference between the infant’s actual age and its average age (‘within-infants age’). Because we predicted differences between the species and assumed that infants would take a more active role throughout ontogeny, we also included four two-way interactions between both species and role with the two variables representing infant age in the model. To control for the effect, we also included infant’s sex and mother’s parity as fixed effects in the model. We included study site and identity of the mother and infant as random effects (intercepts). To keep type 1 error rates at the nominal level of 5%, we also included role, within-infants age, parity and infant sex within subject identity, and site^64^ as random slopes components. We did not include any other random slopes components within mother ID because, with a single exception, each mother was recorded with only a single infant, so the random slopes of these fixed effects within mother ID would be highly redundant with those within infant identity. For the other fixed effects, we did not include random slopes since they were usually constant within mother and infant ID. To keep model complexity at an acceptable level, and because neglected random slopes do not compromise type 1 error rates^64^, we did not include correlations between random slopes and random intercepts.

The models were implemented in R^65^ using the function *glmer* in the package lme4^66^. To test the overall significance of our key test predictors, we used a likelihood ratio test^67^ to compare the full models with a null model that contained only the control predictor with fixed effects and all random effects. If either interaction with within- and between-infants age was non-significant, they were removed from the model. To test whether inter-site differences had a significant effect on the response variables, we excluded site (and all random slopes within site), and ran a second likelihood ratio test comparing the full model with this reduced model. Prior to running the models, we z-transformed between-infants age, within-infants age and parity^68^. To control for collinearity, we determined Variance Inflation Factors (VIF^69^) from a model that included only the fixed main effects, using the function *vif* of the R package car. This revealed that collinearity was not an issue (maximum VIF = 1.23). In the models with Poisson error structure, overdispersion was not an issue (dispersion parameters for distance: 1.03, gesture-response pairs: 0.52). To estimate model stability, we excluded the levels of random effects one at a time, ran the models again and compared the resulting estimates derived with those obtained from the respective models based on all data. This revealed that all models were at least ‘moderately’ stable, particularly for those estimates that were not close to zero. Tests of the individual fixed effects were derived using likelihood ratio tests (R function *drop1* with argument ‘test’ set to “Chisq”). All statistical analyses were performed using R-version R.3.1.1^65^, with the level of significance set to 0.05.

### Ethics statement

Our study was purely non-invasive, with audio and video recordings taken from a minimum distance of seven meters, in an effort to avoid influencing the natural behaviour of the individuals, parties and communities. The research adhered to the legal requirements of the countries in which it was conducted and followed the recommendations of the ‘Animals (Scientific Procedures) Act 1986’, as published by the government of the United Kingdom, and the principles of “Ethical Treatment of Non-Human Primates” as stated by the American Society of Primatologists. Permission to conduct research at the four field sites was granted by the Centre de Recherche en Écologie et Foresterie (CREF; DRC), the Institut Congolaise pour la Conservation de la Nature (ICCN; DRC), the Makerere University Biological Field Station (MUBFS; Uganda), the Ministère de l’Enseignement Supérieure et de la Recherche Scientifique (Côte d’Ivoire), the Ministère de Recherche Scientifique (DRC), the Office Ivoirien des Parcs et Réserves (OIPR; Côte d’Ivoire), the Uganda National Council for Science and Technology (UNCST; Uganda) and the Uganda Wildlife Authority (UWA; Uganda).

## Additional Information

**How to cite this article**: Fröhlich, M. *et al.* Unpeeling the layers of language: Bonobos and chimpanzees engage in cooperative turn-taking sequences. *Sci. Rep.* **6**, 25887; doi: 10.1038/srep25887 (2016).

## Supplementary Material

Supplementary Information

Supplementary Video 1

Supplementary Video 2

Supplementary Video 3

Supplementary Video 4

## Figures and Tables

**Table 1:** Effects of species, role and infant age on the investigated parameters.

	Estimate	se	*χ*^2^	*p*
**1.a Gaze**				
Intercept	−0.121	0.142	^(1)^	^(1)^
** species [chimp]**	−**0.485**	**0.155**	**8.288**	**0.004**
role [mother]	0.108	0.100	1.157	0.282
within-infants age	−0.053	0.050	1.186	0.276
between-infants age	0.075	0.068	1.152	0.283
infant sex [male]	0.040	0.155	0.068	0.794
parity	−0.009	0.080	0.014	0.906
**1.b Body orientation**				
Intercept	0.952	0.177	^(1)^	^(1)^
** species [chimp]**	**0.543**	**0.179**	**4.872**	**0.027**
role [mother]	−1.301	0.117	^(1)^	^(1)^
within-infants age	−0.141	0.134	^(1)^	^(1)^
between-infants age	0.189	0.142	^(1)^	^(1)^
infant sex [male]	0.182	0.186	0.986	0.321
parity	−0.087	0.098	0.753	0.386
role: within-infants age	0.068	0.129	0.275	0.600
** role: between-infants age**	−**0.483**	**0.128**	**16.307**	**<0.001**
**1.c Initiation distance**				
Intercept	0.036	0.097	^(1)^	^(1)^
** species [chimp]**	**0.426**	**0.103**	**7.382**	**0.007**
role [mother]	−0.095	0.078	1.485	0.223
within-infants age	0.106	0.048	2.923	0.087
** between-infants age**	**0.145**	**0.041**	**9.919**	**0.002**
infant sex [male]	−0.080	0.088	0.764	0.382
parity	−0.018	0.046	0.150	0.699
**2.a Gesture-response pairs**				
Intercept	0.135	0.080	^(1)^	^(1)^
species [chimp]	0.129	0.063	^(1)^	^(1)^
** role [mother]**	**0.180**	**0.078**	**5.447**	**0.020**
within-infants age	−0.038	0.078	^(1)^	^(1)^
between-infants age	−0.081	0.065	^(1)^	^(1)^
infant sex [male]	−0.052	0.063	0.693	0.405
parity	−0.007	0.030	0.058	0.810
species: within-infants age	0.057	0.084	0.472	0.492
** species: between-infants age**	**0.179**	**0.071**	**6.381**	**0.012**
**2.b Response waiting**				
Intercept	−2.061	0.177	^(1)^	^(1)^
** species [chimp]**	**0.893**	**0.173**	**19.845**	**<0.001**
role [mother]	0.585	0.137	^(1)^	^(1)^
within-infants age	0.154	0.161	^(1)^	^(1)^
between-infants age	0.422	0.111	^(1)^	^(1)^
infant sex [male]	0.095	0.165	0.327	0.567
parity	0.026	0.082	0.099	0.753
role: within-infants age	−0.234	0.139	2.872	0.090
** role: between-infants age**	−**0.433**	**0.114**	**14.856**	**<0.001**
**3.a Overlapping response**				
Intercept	−0.365	0.142	^(1)^	^(1)^
** species [chimp]**	−**0.650**	**0.127**	**11.656**	**<0.001**
** role [mother]**	**0.382**	**0.135**	**8.256**	**0.004**
within-infants age	−0.091	0.059	1.521	0.217
** between-infants age**	−**0.124**	**0.063**	**3.953**	**0.047**
infant sex [male]	−0.032	0.121	0.068	0.794
parity	−0.038	0.062	0.377	0.540
**3.b Delayed response**				
Intercept	−1.149	0.159	^(1)^	^(1)^
species [chimp]	0.917	0.150	^(1)^	^(1)^
** role [mother]**	−**0.484**	**0.137**	**12.416**	**<0.001**
within-infants age	−0.088	0.227	^(1)^	^(1)^
between-infants age	0.510	0.177	^(1)^	^(1)^
infant sex [male]	−0.004	0.128	0.001	0.975
parity	−0.024	0.063	0.148	0.700
species: within-infants age	0.227	0.242	0.858	0.354
** species: between-infants age**	−**0.510**	**0.187**	**7.564**	**0.006**

^(1)^Not shown as lacking a meaningful interpretation.

Infant sex and parity were included as control predictors; ID and site were included as random effects.

**Table 2:** Details on observed dyads as well as respective age/study period, interaction time and sampling efforts.

Group	Dyad (infant/mother)	Infant sex	Infant age P1 (months)	Infant age P2 (months)	Interaction time (hours)
LuiKotale	Wangila/Wilma	F	14–21	26–28	11.2
	Nora/Nina	F	15–22	28–30	9.1
	Zizu/Zoe	M	22–28	35–37	10.5
	Izzy/Iris	F	26–32	37–40	5.8
	Solea/Susi	F	36–42	48–50	7.3
	Opal/Olga	F	35–42	47–49	8.1
Wamba	Jolie/Jacky	F	09–13	N/A	8.2
	Seko/Sala	M	10–14	N/A	9.5
	Fua/Fuku	F	20–25	N/A	5.9
	Otoko/Otomi	F	21–25	N/A	6.8
	Hachiro/Hoshi	M	38–42	N/A	5.8
	Kiyota/Kiku	M	39– 43	N/A	5.3
Kanyawara	Winza/Wangari	M	09–11	21–23	15.2
	Tembo/Tenkere	M	13–15	25–27	18.4
	Mango/Michelle	F	13–15	25–27	7.3
	Lily/Leona	F	03–05**	15–17	7.2
	Thatcher/Tongo	F	16–18	28–30	15
	Gola/Outamba	F	48–50*	N/A	7.2
	Wallace/Wilma	M	55–57	67–69	10.1
Taï South	Mohan/Mbele	F	10–12	22–24	11.2
	Iniesta/Isha	M	N/A	10-12	12
	Solibra/Sumatra	M	15–17	27–29	14.7
	Jeff/Julia	M	15^†^	N/A	0.4
	Kayo/Kinshasa	F	19–21	31–33	17.0
	Ithaka/Isha	M	64–66*	N/A	9.5
**N**	**25**	**13:12**			**238.7**

P1/P2–first/second period of data collection; ^†^Deceased on Nov 1, 2012; *Mothers gave birth to sibling in P2, thus no P2 data available; **P1 not included.
