# Supplementary material for: Unpeeling the layers of language: Bonobos and chimpanzees engage in cooperative turn-taking sequences
Source: Sci Rep. 2016 May 23;6:25887. doi: 10.1038/srep25887 (PMC4876478; doi:10.1038/srep25887)
Supplement: Supplementary Information [file srep25887-s1.doc]

Unpeeling the layers of language: Bonobos and chimpanzees engage in cooperative turn-taking sequences

Marlen Fröhlich^1^ *, Paul Kuchenbuch^1^, Gudrun Müller^1^, Barbara Fruth^2, 3^, Takeshi Furuichi^4^, Roman M. Wittig^5, 6^, Simone Pika^1^ *

**Supplementary Table S1.** Identified gesture types produced to initiate carries in bonobo (B) and chimpanzee (C) mother-infant dyads at all four study sites. For each gesture type the use by the mother, infant or both is specified. Fraction numbers represent the number of infants, mothers or both (infants + mothers) using the gesture type out of the number of individuals observed at the respective sites.

| **Gesture type** | **Definition** | | | | **Used by** | ***LuiKotale***  **(B)** | ***Wamba***  **(B)** | ***Kanyawara***  **(C)** | ***Taï South* (C)** |  | |
| --- | --- | --- | --- | --- | --- | --- | --- | --- | --- | --- | --- |
| **Audible** |  |  |  |  |  | | | | | |  |
| Slap ground | Signaller hits ground with flat palm of his hand. | | | | Mother | 0/6 | 0/6 | 0/6 | 1/6 |  | |
| Loud scratch | Signaller makes deliberate scratching movements on own body. | | | | Mother | 6/6 | 6/6 | 7/7 | 5/6 |  | |
| **Tactile** |  |  |  |  |  | | | | | |  |
| Arm on | Signaller places palm on recipient’s back (> 2 seconds). | | | | Infant | 0/6 | 1/6 | 2/7 | 1/6 |  | |
| Shake back | Signaller shakes lower back in an upward movement when recipient is already clinging. | | | | Mother | 0/6 | 0/6 | 1/7 | 0/6 |  | |
| Scoop infant | Signaller reaches behind himself and gently pushes infant up onto back with a back ward and upward movement. | | | | Mother | 1/6 | 1/6 | 4/7 | 3/6 |  | |
| Touch | Signaller makes short (> 2 seconds) contact with recipient using palm and/or fingers. | | | | Both | 4/6 + 4/6 | 4/6 + 2/6 | 3/7 + 6/7 | 4/6 + 5/6 |  | |
| Directed push | Signaller uses limbs or body to bring recipient in direction of movement. | | | | Mother | 6/6 | 6/6 | 5/7 | 5/6 |  | |
| Pull | Signaller moves recipient’s body part towards himself. | | | | Both | 0/6 + 5/6 | 1/6 + 5/6 | 1/7 + 4/7 | 0/6 + 4/6 |  | |
| **Visual** |  |  |  |  |  | | | | | |  |
| Backward sweep | Signaller stretches arm towards behind himself in a short, rapid movement. | | | | Mother | 1/6 | 0/6 | 2/7 | 0/6 |  | |
| Extend leg/ARM | Signaller extends leg to facilitate climb onto self. | | | | Mother | 3/6 | 4/6 | 5/7 | 3/6 |  | |
| Look | Signaller gazes at recipient (> 2 seconds). | | | | Both | 3/6 + 6/6 | 5/6 + 5/6 | 2/7 + 6/7 | 4/6 + 5/6 |  | |
| Lower back | Signaller, in lateral position to recipient, lowers abdomen without stopping locomotion. | | | | Mother | 1/6 | 0/6 | 1/6 | 1/6 |  | |
| Stop and look back/down | Signaller stops with body orientated in direction of movement and looks back (or down) at recipient. | | | | Both | 1/6 + 6/6 | 3/6 + 5/6 | 1/7 + 6/7 | 2/6 + 6/6 |  | |
| Present back/venter | Signaller offers back/venter to recipient. | | | | Mother | 4/6 | 5/6 | 7/7 | 5/6 |  | |
| Reach | Signaller extends arm toward recipient. | | | | Both | 3/6 + 5/6 | 4/6 + 4/6 | 2/7 + 5/6 | 3/6 + 3/6 |  | |
| Rear up | Signaller briefly rises straight up on two feet while positioned towards recipient. | | | | Mother | 0/6 | 0/6 | 0/7 | 2/6 |  | |
| Turn Bipedal | Signaller turns towards recipient with short bipedal movement | | | | Mother | 0/6 | 0/6 | 0/7 | 1/6 |  | |

Supplementary Table S2. Distribution of observed joint-travel interactions with regard to species (bonobo, chimpanzee), study site (LuiKotale, Wamba, Kanyawara, Taï), dyad (total number) and initiator in dyad (mother, infant).

| **SITE** | **CARRY INITIATOR** | | |  |
| --- | --- | --- | --- | --- |
| **Bonobos** | **Mother** | **Infant** | **Undecided** | **Total** |
| **LuiKotale** | **69** | **52** | **34** | **155** |
| IRIZ | 6 | 11 | 7 | 24 |
| NINO | 11 | 5 | 5 | 21 |
| OLOP | 6 | 10 | 5 | 21 |
| SUSO | 11 | 10 | 2 | 23 |
| WIWG | 10 | 6 | 6 | 22 |
| ZOZE | 25 | 10 | 9 | 44 |
| **Wamba** | **95** | **43** | **26** | **164** |
| FKFA | 14 | 12 | 5 | 31 |
| HSHC | 4 | 6 | 4 | 14 |
| JKJL | 25 | 9 | 6 | 40 |
| KIKT | 8 | 8 | 2 | 18 |
| OTOK | 13 | 5 | 6 | 24 |
| SOSU | 31 | 3 | 3 | 37 |
| **Chimpanzees** | **Mother** | **Infant** | **Undecided** | **Total** |
| **Kanyawara** | **153** | **62** | **3** | **218** |
| LNLL | 12 | 4 | 1 | 17 |
| MLMM | 23 | 1 | 0 | 24 |
| OTOB | 26 | 11 | 0 | 37 |
| OUOL | 0 | 10 | 1 | 11 |
| TGTR | 29 | 11 | 0 | 40 |
| WAWZ | 43 | 4 | 0 | 47 |
| WLWC | 20 | 21 | 1 | 42 |
| **Taï South** | **119** | **74** | **4** | **197** |
| ISIN | 18 | 10 | 2 | 30 |
| ISIT | 1 | 5 | 0 | 6 |
| JLJF | 7 | 3 | 0 | 10 |
| KSKY | 36 | 18 | 1 | 55 |
| MBMH | 38 | 11 | 1 | 50 |
| SMSL | 19 | 27 | 0 | 46 |
| **Grand Total** | **436** | **231** | **67** | **734** |

Supplementary Figure S1. Frequency distribution of response times (in bins of 200 milliseconds) after joint travel initiation gestures in relation to species. Arrows depict medians for each species (bonobos: *Md* = 1000 ms, *N* = 952; chimpanzees: *Md* = 1400 ms, *N* = 1815).

**Video clip Legends**

**Video S1**: Bonobo mother initiating joint travel

**Video S2**: Bonobo infant initiating joint travel

**Video S3**: Chimpanzee mother initiating joint travel

**Video S4**: Chimpanzee infant initiating joint travel
